# Expression of concern: Overexpression of long intergenic noncoding RNA LINC00312 inhibits the invasion and migration of thyroid cancer cells by down-regulating microRNA-197-3p

**DOI:** 10.1042/BSR-20170109_EOC

**Published:** 2020-08-21

**Keywords:** Cell migration, invasion and migration, Long intergenic noncoding RNA LINC00312, MicroRNA-197-3p, proliferation, Thyroid cancer

The Editorial Office has been made aware of potential issues surrounding the scientific validity of this paper, hence has issued an expression of concern to notify readers whilst the Editorial Office investigates.

